# Parametric Study and Electrocatalyst of Polymer Electrolyte Membrane (PEM) Electrolysis Performance

**DOI:** 10.3390/polym15030560

**Published:** 2023-01-21

**Authors:** Adam Mohd Izhan Noor Azam, Ng Khai Li, Nurul Noramelya Zulkefli, Mohd Shahbudin Masdar, Edy Herianto Majlan, Nurul Akidah Baharuddin, Azran Mohd Zainoodin, Rozan Mohamad Yunus, Noor Shahirah Shamsul, Teuku Husaini, Siti Nur Amira Shaffee

**Affiliations:** 1 Fuel Cell Institute, Universiti Kebangsaan Malaysia, Bangi 43600, Selangor, Malaysia; 2 Department of Chemical & Process Engineering, Faculty of Engineering & Built Environment, Universiti Kebangsaan Malaysia, Bangi 43600, Selangor, Malaysia; 3 Petronas Research Sdn Bhd (PRSB), Jalan Ayer Hitam, Kawasan Institusi Bangi, Bandar Baru Bangi 43650, Selangor, Malaysia

**Keywords:** polymer electrolyte membrane (PEM) electrolyzer, water electrolysis, parametric study, energy efficiency, electrochemical reaction

## Abstract

An investigation was conducted to determine the effects of operating parameters for various electrode types on hydrogen gas production through electrolysis, as well as to evaluate the efficiency of the polymer electrolyte membrane (PEM) electrolyzer. Deionized (DI) water was fed to a single-cell PEM electrolyzer with an active area of 36 cm^2^. Parameters such as power supply (50–500 mA/cm^2^), feed water flow rate (0.5–5 mL/min), water temperature (25−80 °C), and type of anode electrocatalyst (0.5 mg/cm^2^ PtC [60%], 1.5 mg/cm^2^ IrRuOx with 1.5 mg/cm^2^ PtB, 3.0 mg/cm^2^ IrRuOx, and 3.0 mg/cm^2^ PtB) were varied. The effects of these parameter changes were then analyzed in terms of the polarization curve, hydrogen flowrate, power consumption, voltaic efficiency, and energy efficiency. The best electrolysis performance was observed at a DI water feed flowrate of 2 mL/min and a cell temperature of 70 °C, using a membrane electrode assembly that has a 3.0 mg/cm^2^ IrRuOx catalyst at the anode side. This improved performance of the PEM electrolyzer is due to the reduction in activation as well as ohmic losses. Furthermore, the energy consumption was optimal when the current density was about 200 mA/cm^2^, with voltaic and energy efficiencies of 85% and 67.5%, respectively. This result indicates low electrical energy consumption, which can lower the operating cost and increase the performance of PEM electrolyzers. Therefore, the optimal operating parameters are crucial to ensure the ideal performance and durability of the PEM electrolyzer as well as lower its operating costs.

## 1. Introduction

Hydrogen (H_2_) has been identified and recognized as a potentially clean, sustainable, and storable alternative energy source. It is environmentally friendly and does not release harmful gases [1,2]. Moreover, it can supply two to three times the energy of other alternative fuels, such as ethanol, methanol, biodiesel, liquefied petroleum gas, and natural gas. Thus, hydrogen has the potential to replace or minimize gasoline consumption [3,4]. Various methods of producing hydrogen from water are also available, such as thermochemical methods, thermolysis, photolysis, and electrolysis. Among the available methods, water electrolysis is the most environmentally friendly and produces ultrapure hydrogen gas (99.999%). In water electrolysis, a direct current is applied across electrodes during electrolysis to provide a potential gradient and driving force for ion diffusion in the cell, which then splits water into hydrogen gas (cathode) and oxygen gas (anode) [5,6,7,8]. Electrolyzed hydrogen can be utilized as an energy and power source for hydrogen fuel cells, whereas oxygen can be used in various industrial processes or as a gas in the medical field [9].

Three types of electrolyzers are currently available in the market, namely, alkaline electrolyzers, solid oxide electrolyzers, and polymer electrolyte membrane (PEM) electrolyzers. Of the three electrolyzer types, PEM electrolyzers are commercially available and are commonly used for producing “green-hydrogen” from renewable energy sources due to their efficiency and adaptability [10,11]. PEM electrolyzers can operate at high current densities (above 2 A/cm^2^) with high voltage efficiency. Other advantages of PEM electrolyzers are the capacity to produce hydrogen with high purity, operate under high-pressure conditions, and rapidly store hydrogen fuel [12,13]. Compared to others, PEM electrolyzers produce hydrogen with higher impermeability and higher proton conductivity.

The challenge of using commercial PEM electrolyzers is the high component cost of the cell. Their membrane electrode assembly (MEA) component, iridium, is the rarest element in Earth’s crust. It also has other applications. Thus, the high demand for iridium increases its cost. Moreover, iridium oxide exhibits low activity, and ruthenium oxide has low corrosion resistance, both of which affect the performance of PEM electrolyzers in oxygen evolution reactions (OER). These limitations affect the durability of PEM electrolyzers and lead to expensive and significant maintenance costs [14,15,16]. Therefore, the difference in the operating conditions of PEM electrolyzers and the type of electrocatalyst used through experimental or model development are crucial for improving PEM electrolyzer performance and lifespan to lower operating costs.

Various studies have been conducted on the performance variance of PEM water electrolysis. Many of these studies have shown that operation parameters such as current density, flow field, pressure, temperature, and others can affect the performance of a PEM water electrolyzer. Tijani et al. [17] developed a simulation model to investigate the effects of the exchange current density and charge-transfer coefficient (CTC) on the performance of PEM electrolyzers. They observed that the input power increases exponentially with increasing current. In addition, analysis of the effect of temperature on the polarization curve showed a significant decrease in the operating voltage in the 20 °C to 80 °C temperature range. Selamet et al. [18] conducted a study using 100 cm^2^ single-cell and 10-cell stack PEM electrolyzers to determine the effects of feed water flowrate, temperature, and pressure on cell performance. The results showed that performance was enhanced at higher operating temperatures and low pressure. They concluded that the water flowrate indirectly affects the performance of PEM electrolysis even though the results are insignificant.

Meanwhile, Majasan et al. [19] used high-speed optical imaging to study the two-phase anode flow-field flow behavior and gas-bubble dynamics of a PEM electrolyzer under various operating settings and link the results to electrochemical performance. They showed that a parallel flow field yielded better performance than a serpentine flow field. The effect of water flow rate on performance depends strongly on the cell operating temperature, and higher water flow rates delay bubbly-to-slug transition and lead to the formation of smaller bubbles and shorter slugs. In studies conducted by Araya et al. [20], a seven-cell PEM water electrolysis stack was built and tested under various operating conditions. The voltage change and polarization curves were recorded under various test settings, including current density, temperature, and pressure. The results showed that the operating temperature had more obvious effects on the stack performance than the current density and cathode pressure.

Xu and Scott [21] found that the Nafion ionomer contents of the anode and cathode of MEAs affect the performance of single-cell PEM electrolyzers; they measured a current density of 1 A/cm^2^ when the terminal voltage was 1.586 V, the temperature was 80 °C, and Nafion 117, Pt/C was used as the cathode and Ru_0.7_Ir_0.3_O_2_ was the anode. In a study by Rozain et al. [22], the effect of iridium oxide loadings at the anode of a PEM electrolyzer on the overall electrochemical performance was studied using cyclic voltamperometry and electrochemical impedance spectroscopy, under operating conditions of 80 °C and atmospheric pressure and using a 25 cm^2^ single cell. They found that the cell voltage gradually decreased as the anodic loading increased until a value of approximately 1 mg/cm^2^ IrO_2_ was reached; these results are due to improved OER kinetics. According to research conducted by Siracusano et al. [23], decreasing the Ir_0.7_Ru_0.3_Ox loading from 1.5 mg/cm^2^ to 0.5 mg/cm^2^ caused an increase in overpotential and modified the electronic configurations of iridium and ruthenium sites on the surface. The stability for the MEA with a total noble metal catalyst loading of 1.6 mg/cm^2^ was excellent when steady-state durability tests were performed for 1000 h at 1 A/cm^2^.

Despite the tremendous research on the operating conditions of PEM electrolysis, the conditions for the long-term application of this method have yet to be determined as most researchers focus only on the cell voltage and CTC to identify the optimum operating conditions. Thus, this research focuses on the effects of operating conditions such as current density, water flowrate, operating temperature and electrode type, i.e., electrocatalyst type and electrocatalyst loading, on the performance of the electrolyzer, particularly in terms of hydrogen production, cell electrical energy consumption, voltaic efficiency, and energy efficiency, to reduce the operating costs.

## 2. Methodology

### 2.1. Single-Cell PEM Electrolyzer

**Figure 1:**
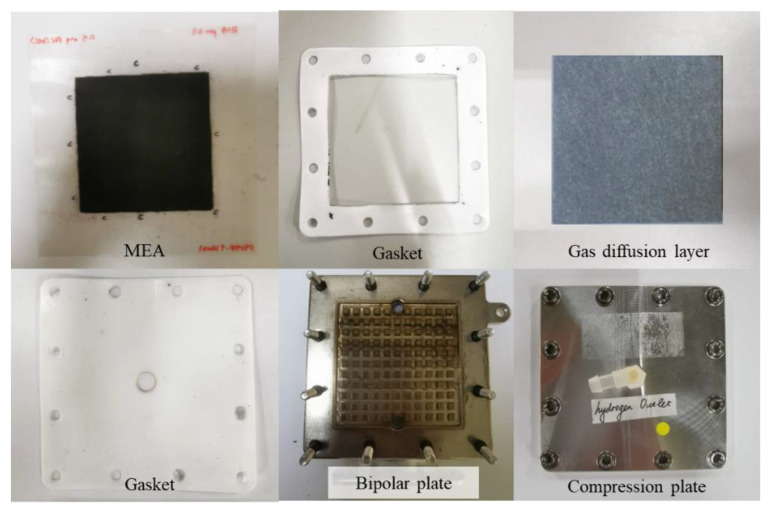
Components of a single-cell stack electrolyzer.

A single-cell PEM electrolyzer was used throughout the studies. A PEM electrolyzer consists of several basic components, including an MEA, which is the core of a cell, and other components such as a gas diffusion layer, bipolar plate, gaskets, and a compression plate. The MEA, which has an active area of 36 cm^2^ (6 cm × 6 cm) and anode and cathode catalysts, is the main component. Nafion 115 was used as the MEA electrolyte membrane. The MEA is reinforced with two bipolar plates. Platinum coated titanium plate (1 um in thickness) was used as a bipolar plate. The anode flow field design for bipolar plate was pin type, while plane bipolar plate with titanium felt was used at the cathode side. Between the MEA and the bipolar plate are layers of gaskets whose number depends on the desired dimensions. Figure 1 shows the single-cell components of the PEM electrolyzer. Table 1 shows the specifications for the different types of MEA used.

**Figure 2:**
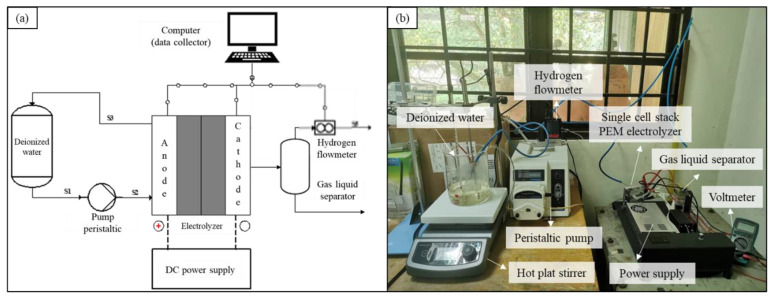
(**a**) Schematic diagram and (**b**) actual photo of the experimental setup.

A single-cell stack was connected to the peristaltic pump, hydrogen flowmeter, and power supply. Figure 2a,b show the schematic diagram and actual photo of the experimental setup in this study, respectively.

### 2.2. Operation of the PEM Electrolyzer

The performance of the PEM electrolyzer in the production of hydrogen gas under the different power supplies, water feed flowrate, temperature conditions, and types of anode electrocatalysts was evaluated. Deionized (DI) water was fed into the anode side of the single-cell stack at the desired flowrate using a peristaltic pump to ensure the flow of water into the PEM electrolyzer. The DI water was heated, and its temperature was maintained at the desired point as it was fed into the electrolyzer at a constant flowrate for 30 min. The experiment was conducted by varying the current density within the 50 mA/cm^2^ to 500 mA/cm^2^ range. The cell voltage was measured and recorded after 30 min to plot a polarization curve (also known as a *V*−*I* curve). The flowrate of hydrogen produced through the PEM electrolyzer was also recorded.

The flowrate was varied within the 0.5 mL/min to 5 mL/min range by keeping the temperature constant at 30 °C. The experiment was repeated by varying the operating temperature from 25 °C to 80 °C while the water flowrate was maintained at 2 mL/min. A commercial MEA was used when these two parameter modifications were performed. Lastly, the effects of the type of anode electrocatalyst were investigated by using an MEA that has a Nafion^®®^115 membrane as the electrolyte, and the anode side of the electrolyzer was covered with different electrocatalysts, such as 0.5 mg/cm^2^ PtC (60%), 1.5 mg/cm^2^ IrRuOx with 1.5 mg/cm^2^ PtB, 3.0 mg/cm^2^ IrRuOx, and 3.0 mg/cm^2^ PtB. At the same time, 3.0 mg/cm^2^ of PtB was applied on the cathode side of all MEAs used as the cathode catalyst.

### 2.3. Determination of PEM Electrolyzer Performance

The performance of the PEM electrolyzer was evaluated according to the energy consumption, voltaic efficiency, and electrical efficiency based on the cell voltage and amount of hydrogen produced. The equation that was used to determine the energy consumption to produce 1 kg of hydrogen is shown below.

(1) $$E=\frac{I_{\mathrm{cell}}{\;\times \;V}_{\mathrm{cell}}}{F_{H_{2}}{\times \;\rho }_{H_{2}}}$$

where:

*E* = electrical energy consumption (kWh/kg H_2_)
*I*_cell_ = cell current (A)
*V*_cell_ = cell voltage (V)
$F_{H_{2}}$ = hydrogen flowrate (L/h)
$\rho_{H_{2}}$ = density of hydrogen (0.0813 kg/m^3^) at 1 bar, 25 °C

The standard electrochemical potential that corresponds to a high heating value (HHV) is 1.48 V/cell. This value represents a thermoneutral voltage at which hydrogen and oxygen are produced with 100% thermal efficiency. It is the voltage required to split liquid water and the voltage at which a cell operating at 25 °C can operate without producing excessive heat. Therefore, the formula for calculating the cell voltaic efficiency is as follows.

(2) $$\eta =\frac{V_{\mathrm{TN}}}{V_{\mathrm{cell}}}$$

where:

*η* = cell voltaic efficiency (%)
*V_TN_* = thermoneutral voltage = 1.48 V
*V_cell_* = cell voltage

While the cell operates, an electrochemical reaction draws heat from the cell components and cools the cell until it stops operating. Therefore, to maintain temperature, sensible heat must be supplied to the cell components from an external source. In the case of global system efficiency, both electrical input and heat input from external sources must be included. Energy efficiency can be calculated using the following equation:

(3) $$\mathrm{energy}\;\mathrm{efficiency}\;(\%)=\frac{{\mathrm{HHV}\;\mathrm{of}\;H}_{2}\;\mathrm{production}}{{\mathrm{electrical}\;\mathrm{energy}\;\mathrm{consumption}\;(\mathrm{kWh}/\mathrm{kg}\;H}_{2})}\;\times \;100$$

where HHV of H_2_ production = 39.4 kWh/kg.

## 3. Results and Discussion

### 3.1. Effect of Water Feed Flowrate

**Figure 3:**
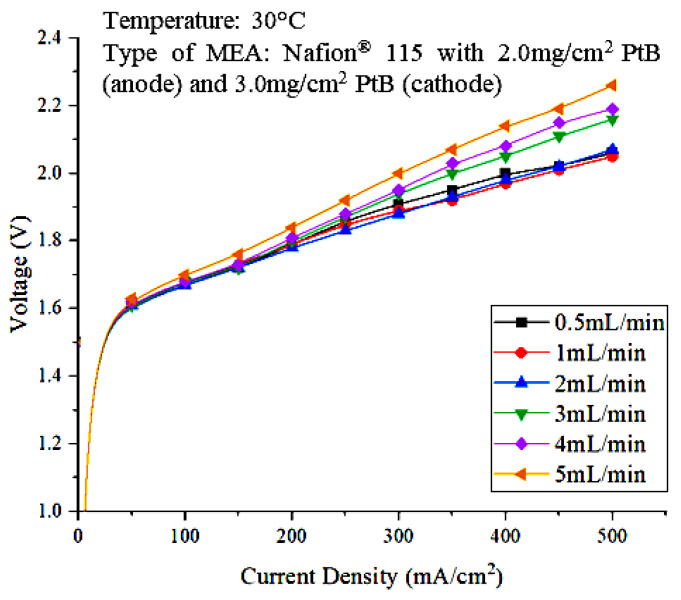
Polarization curve representing the effect of water feed flowrate on PEM electrolyzer performance.

The effect of the water feed flowrate was determined to identify the most optimal water feed flowrate for operating the PEM electrolyzer. The power supply was varied within the 50 mA/cm^2^ to 500 mA/cm^2^ range. The feed water flowrates used in this study ranged from 0.5 mL/min to 2 mL/min. A polarization curve, which is a graph that shows the relationship between cell voltage and current density, was then plotted. Figure 3 shows the polarization curve that represents the effect of water flowrate on PEM electrolyzer performance.

**Figure 4:**
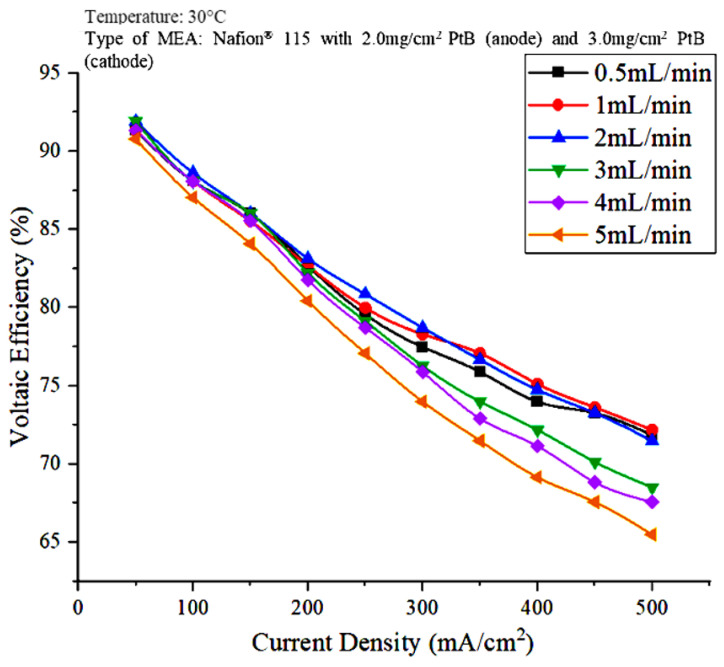
Effect of water flowrate on voltaic efficiency.

Based on Figure 3, the cell voltages at different current densities are nearly the same for the 0.5, 1, 2, 3, and 4 mL/min flowrates at low current densities of 50 mA/cm^2^ to 200 mA/cm^2^. However, a clear difference can be seen as the current density increases. Cell performance was better for the 2 mL/min water flowrate compared to other flowrates when the current density was higher, as it contributed to lower cell voltages. This result is reasonable because higher current densities can increase the reaction kinetics at both electrodes and thus lead to lower charge-transfer resistance, which contributes to better cell performance [20]. Therefore, based on the polarization curve, the optimal flowrate is 2 mL/min. The flowrate of water fed into the water electrolysis unit can affect the rate of hydrogen production. Figure 4 shows the performance of the PEM electrolyzer as evaluated in terms of voltaic efficiency.

The voltaic efficiency is calculated through the electrochemical potential that corresponds to the HHV, which is equivalent to 1.48 V. The voltage efficiency decreased as the current density increased because, according to the equation for voltage efficiency, the cell voltage is inversely proportional to the voltage efficiency. Figure 4 shows that the voltage efficiency at a water flowrate of 2 mL/min and a current density of 200 mA/cm^2^ is the highest at 83.15%.

**Figure 5:**
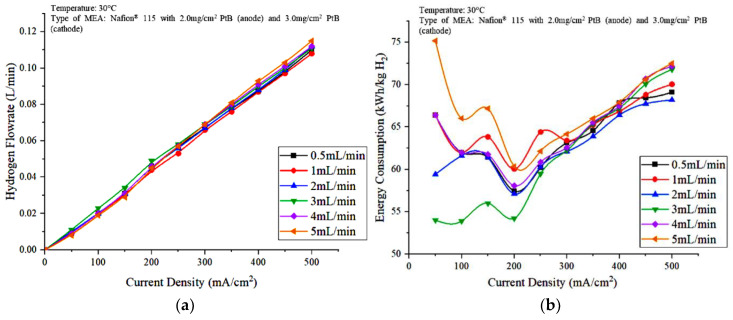
Effects of water flowrate on the (**a**) hydrogen production and (**b**) energy consumption of the PEM electrolyzer.

Figure 5 shows the effect of water flow rate on the hydrogen production and the energy consumption against current density (Figure 5) was constructed to study the effect of the water flowrate on the PEM electrolyzer performance. Based on Figure 5a, the significant difference in the hydrogen production under different water flow rate conditions is not clearly shown. When water was fed into the PEM electrolyzer at 3 mL/min, the hydrogen produced was slightly higher at densities between 50 and 300 mA/cm^2^ compared to the other DI water flow rate. At current densities greater than 300 mA/cm^2^, the hydrogen flowrate increased as the water flowrate increased from 1 mL/min to 5 mL/min. However, overall, the water feed flowrate of 3 mL/min is the optimal condition for hydrogen production using an PEM electrolyzer. Although 3 mL/min can be said to be optimal in terms of hydrogen gas production, the energy consumption from 250 mA/cm^2^ to 500 mA/cm^2^ increased based on Figure 5b. Figure 5b also shows that the consumption of electricity was the highest at 5 mL/min water flowrate compared with those at other water flowrates. This result is due to the role of water as a cooling agent, which means that less heat is supplied through the higher enthalpy of the water flow and results in the availability of less efficient reactants to the anode electrodes [19]. The water flowrate at 2 mL/min is arguably optimal after 250 mA/cm^2^ due to the low energy consumption. The cell voltage at a low 2 mL/min water flowrate is directly proportional to the electrical energy consumption of the PEM electrolyzer, which leads to less electrical energy used for cell operation compared with those at other feed water flowrates. The lowest energy consumption is about 54.22 kWh/kg H_2_ when the flowrate was 3 mL/min and a current of 7.2 A was supplied to the electrolysis unit.

**Figure 6:**
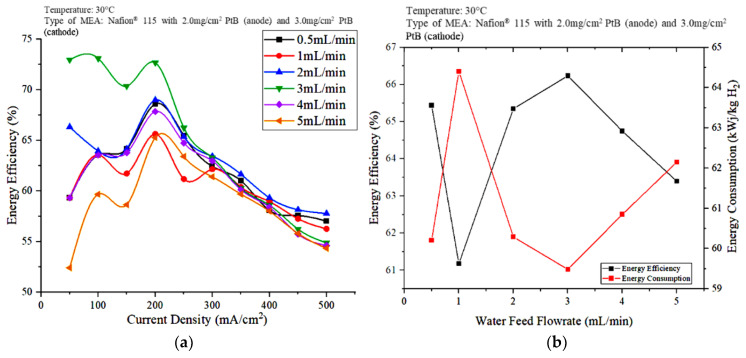
Effects of water flowrate on PEM electrolyzer performance (**a**) energy efficiency versus current density (**b**) energy efficiency versus water flow rate.

The performance of the electrolyzer was further evaluated in terms of energy efficiency, as shown in Figure 6. Based on Figure 6a, the energy efficiency shows a fluctuating trend at current densities of less than 250 mA/cm^2^. When the current density was less than 250 mA/cm^2^, the water flowrate at 3 mL/min resulted in the highest energy efficiency. However, the energy efficiency shows a downward trend for 3 mL/min at current densities greater than 300 mA/cm^2^. At this point, i.e., above 300 mA/cm^2^, 2 mL/min showed lower electrical energy consumption as result in highest energy efficiency. Meanwhile, Figure 6b shows the plot of the energy efficiency and energy consumption against the feedwater flowrate at a constant current density of 250 mA/cm^2^.

When the current density was kept constant at 250 mA/cm^2^ and the water flowrate was 3 mL/min, the hydrogen produced was highest, and the energy consumption was low. Meanwhile, electricity consumption shows no definite trend against water flowrate, whereas the energy consumption and hydrogen production increased in the water flowrate order of 3, 0.5, 2, and 4 mL/min, followed by 5 mL/min when the current density was kept constant. The performance of the PEM electrolyzer was also evaluated in terms of energy efficiency. As shown in Figure 6b, the water flowrate of 3 mL/min resulted in the lowest energy consumption with the highest energy efficiency. The difference between the energy efficiencies obtained at 2 and 3 mL/min when the current density was 250 mA/cm^2^ is small. However, the performance of the PEM electrolysis unit is evaluated, not only in terms of energy efficiency and energy consumption, but also in terms of voltage efficiency, hydrogen flowrate, energy consumption, and energy efficiency at different power supply voltages.

Overall, the water flowrate can affect the performance of the electrolysis unit in various aspects. Based on the results obtained, 2 mL/min is the optimal water flowrate for operating the PEM electrolyzer. According to a study by Choi et al. [24], the cooling rate increases as the flowrate increases. Therefore, the operating temperature of a single cell decreases. This result means that the outlet temperature at the anode part of the cell is low when the flowrate is high. This phenomenon reduces ion conductivity and causes the activation overpotential to increase with decreasing operating temperature. Therefore, in the current study, the water flowrate was kept constant at 2 mL/min to study the effect of the operating temperature and type of electrode catalyst on the PEM electrolyzer.

### 3.2. Effect of Operating Temperature

**Figure 7:**
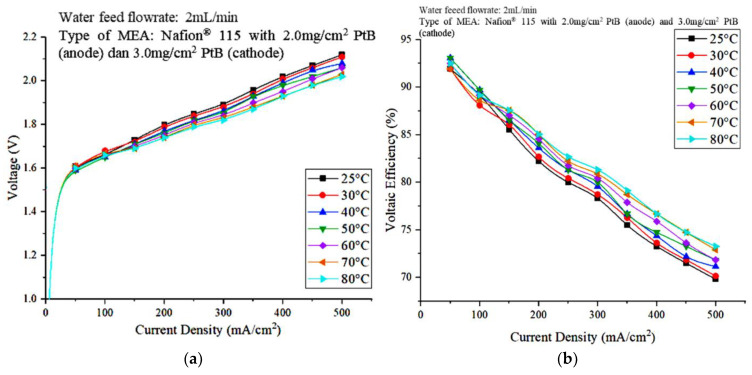
Effect of the operating temperature on PEM electrolyzer performance (**a**) Polarization curves (**b**) Voltaic efficiency.

To identify the effect of the operating temperature on the performance of water electrolysis PEM and determine the optimal operating temperature for the PEM electrolyzer, the effect of the operating temperature was investigated by heating the water at temperatures that range from 25 °C to 80 °C. Figure 7a,b show the polarization curve and (b) voltaic efficiency, respectively, that represent the effect of the operating temperature on PEM electrolyzer performance.

The polarization curve in Figure 7a shows that the voltage decreased as the temperature of the DI water increased. It shows that the operating temperature of 80 °C recorded a low cell voltage value. This result suggests that the higher temperature of 80 °C helped increase the rate of electrocatalytic hydrogen evolution and oxygen evolution at both electrodes and reduced the charge-transfer resistance on both sides [20]. Therefore, the kinetics of the charge-transfer reaction improved at the electrode membrane interface, which led to better cell performance at higher temperatures. The activation overpotential decreases with increasing operating temperature [24]. In addition, the conductivity of the proton exchange membrane increases with increasing temperature, which results in lower ohmic overvoltage at higher temperatures [25].

Moreover, the polarization curve in the lower current density region is steeper than that in the higher current region. This result may be due to the reaction kinetics dominating the entire stack process at lower current density areas, and the charge-transfer resistance is high when reaction kinetics are lower, which, in turn, results in a steeper polarization slope. The higher current density reduced the charge-transfer resistance on both sides, and the ohmic resistance dominated the high-current density area. Therefore, the slope of the polarization curve in the area of high current density is smaller. Meanwhile, in Figure 7b, the lowest voltage efficiency is 25 °C, and the value increased when the operating temperature was increased. This result is due to the increased voltage efficiency when the cell voltage decreased. A voltage efficiency of 85% was achieved when the operating temperature and current density were 80 °C and 200 mA/cm^2^, respectively.

**Figure 8:**
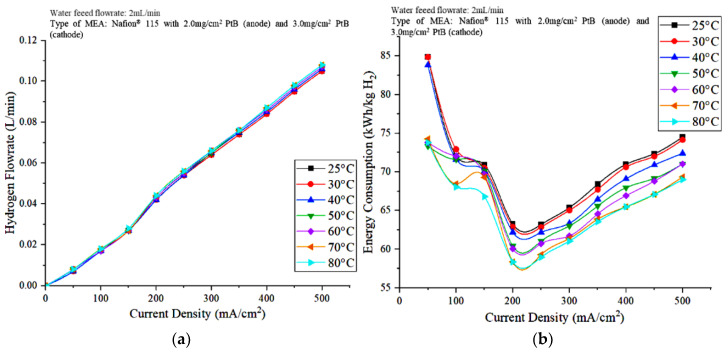
Effects of the operating temperature on the (**a**) hydrogen production and (**b**) energy consumption of the PEM electrolyzer.

Figure 8a,b show the effects of the operating temperature of the PEM electrolyzer on hydrogen production rate and energy consumption, respectively. The hydrogen flowrate increased as the current supplied to the PEM electrolyzer increased. An increase in the current density results in the availability of the overpotential for the subsequent process, leading to a faster breakdown of water molecules in hydrogen and oxygen compared to those in lower current densities [26]. According to Figure 8a, the hydrogen flowrate increased with increasing operating temperature. An increase in cell temperature results in an increase in cell performance as a result of a decrease in cell polarization at a certain current. In turn, these phenomena result in less power consumption for certain hydrogen production rates. Energy consumption can be calculated from the voltage and hydrogen flowrates of the PEM electrolyzer. The optimal energy consumption was observed when the current density was 200 mA/cm^2^ for all operating temperatures, and the energy consumption decreased with increasing stack temperature.

**Figure 9:**
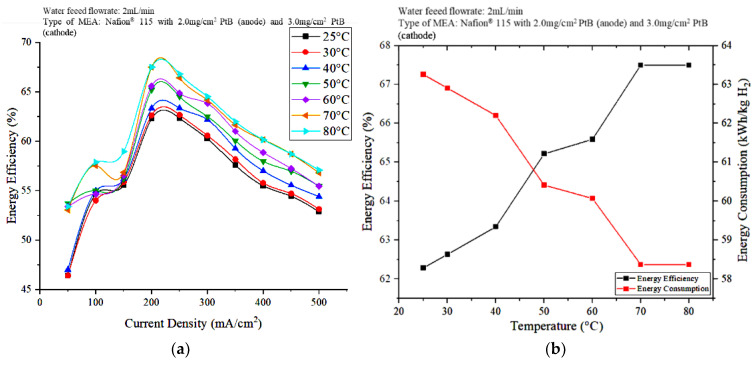
Effects of the operating temperature on (**a**) energy efficiency at different current densities and (**b**) energy efficiency and energy consumption at 200 mA/cm^2^.

Figure 9 shows the effect of the operating temperature on energy efficiency. As can be seen in Figure 9a, the energy efficiency shows an increasing trend as the operating temperature increases, at different applied current densities. This result indicates that the energy efficiency reached its peak of about 67.5% at 80 °C and 200 mA/cm^2^. The hydrogen flowrate increased as the operating temperature increased, indicating that the optimal operating temperatures to achieve the best PEM electrolyzer performance are 70 and 80 °C. At 70 and 80 °C, the energy consumption is the minimum at 58.37 kWh/kg H_2_, whereas at 25 °C, higher energy of 63.26 kWh/kg H_2_ was used at 200 mA/cm^2^ (Figure 9b). The low energy consumption means that the operating cost of the PEM electrolyzer can be reduced; this reduction meets the objectives of this study.

The performance of the PEM electrolyzer was also evaluated in terms of energy efficiency. When the current density was 200 mA/cm^2^, the energy efficiency and energy consumption at 70 and 80 °C are nearly the same at 67.5% and 58.37 kWh/kg H_2_, respectively. However, at 80 °C, the PEM electrolyzer performed well in terms of voltage efficiency and hydrogen production. Hence, the differences in the voltage efficiency and hydrogen production rate observed at 70 and 80 °C are less significant. The use of electrical energy for heating DI water should be emphasized; that is, the higher electrical energy required to heat water to 80 °C compared to heating to 70 °C is relatively insignificant because, as shown in Figure 9b, the electrical energy consumption and energy efficiency achieved by the cell are the same when the current density is 200 mA/cm^2^. Therefore, the optimal operating temperature and power supply are 70 °C and 200 mA/cm^2^ current density.

### 3.3. Effect of Electrode Type

**Figure 10:**
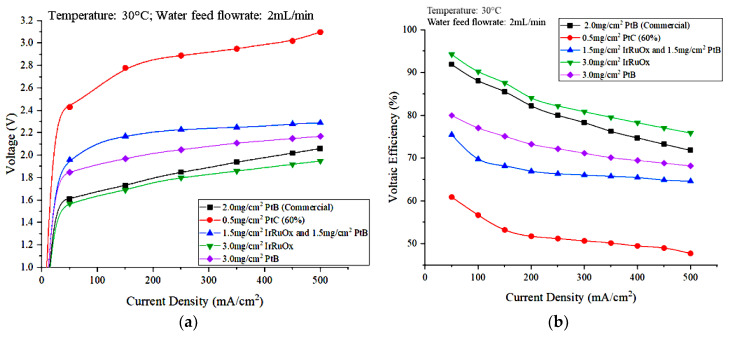
Effects of the anode electrocatalyst type and loading on electrolyzer performance: (**a**) polarization curve that represent the effects and (**b**) effect on voltaic efficiency.

The effects of the type and loading of the electrocatalyst were studied to identify the appropriate type of catalyst and ensure the optimal operation of the PEM electrolyzer. The types and loadings of the electrode catalyst used at the anode side were 0.5 mg/cm^2^ PtC (60%), 1.5 mg/cm^2^ IrRuOx with 1.5 mg/cm^2^ PtB, 3.0 mg/cm^2^ IrRuOx, and 3.0 mg/cm^2^ PtB. The catalyst for the cathode part, 3.0 mg/cm^2^ PtB, was the same for all experiments. The effects of electrode catalyst type and loading on the flowrate of hydrogen generated from water electrolysis, energy consumption, voltaic efficiency, and energy efficiency are discussed, along with the polarization curves that represent these effects. Figure 10a,b show the polarization curves and the effect of the anode electrocatalyst type and loading on voltaic efficiency, respectively.

Figure 10a shows that the highest cell voltage value was achieved using the MEA, which has the lowest electrocatalyst load of 0.5 mg/cm^2^ PtC (60%) at the anode side. For MEAs with a catalyst loading of 3 mg/cm^2^, the voltage obtained using an IrRuOx catalyst was lower than that obtained using PtB. The MEA that has 1.5 mg/cm^2^ IrRuOx and 1.5 mg/cm^2^ PtB produced higher voltage than the 3 mg/cm^2^ PtB. According to a study by Stefania et al. [23], a decrease in the load of the anode catalyst results in a significant increase in polarization resistance due to changes in the charge-transfer resistance associated with oxygen evolution. A decrease in anode load has a greater effect, particularly in the activation region at low current densities. This result is due to the decreased number of catalyst sites when thinner electrodes are used [23].

The use of IrO_2_ as a catalyst in PEM electrolyzers is limited by its high cost and relatively low activity for oxygen evolution., However, high RuO_2_ activity and high IrO_2_ stability can be maintained by using a combination of RuO_2_ and IrO_2_. Thus, a better electrolysis performance was observed for the MEA using 3.0 mg/cm^2^ IrRuOx compared with that using 3.0 mg/cm^2^ PtB. Conductive carbon such as carbon black, nanofibers, or nanotubes that are commonly used as catalyst supports undergo rapid electrochemical oxidation at high anode potentials in PEM electrolysis [27]. Therefore, long-term use of carbon materials as catalyst supports is not possible. To use nanostructured catalysts (*d* < 10 nm) efficiently, other supporting materials that are more resistant to oxidation are required. Moreover, the lower electrocatalytic activity of Pt is due to the high-resistance oxide film that forms on the Pt surface. By contrast, RuO_2_ and IrO_2_ exhibit high electronic conductivity [28]. This ability reduces the overpotential of the cell and can improve the performance of the PEM electrolyzer.

Meanwhile, Figure 10b shows that the cell voltage efficiencies of the different anode electrocatalysts are in the order of 0.5 mg/cm^2^ PtC (60%) > 1.5 mg/cm^2^ IrRuOx and 1.5 mg/cm^2^ PtB > 3.0 mg/cm^2^ PtB > 3.0 mg/cm^2^ IrRuOx. This result indicates that the voltaic efficiency is influenced by the cell voltage.

**Figure 11:**
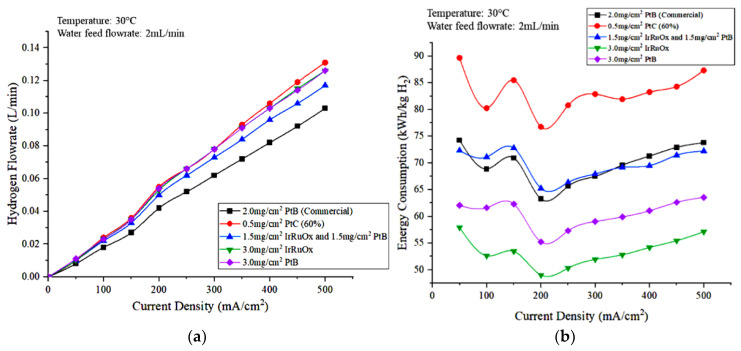
Effects of anode electrocatalyst type and loading on the (**a**) hydrogen production and (**b**) energy consumption of the PEM electrolyzer.

Figure 11a,b show the hydrogen flowrate and energy consumption, respectively, observed for the different types and loading of electrode catalysts. In terms of hydrogen production, commercial MEA produces the lowest hydrogen volume. Figure 11a shows that the hydrogen flowrates measured using 3.0 mg/cm^2^ IrRuOx and 3.0 mg/cm^2^ PtB are nearly the same, and no highly significant difference was observed when compared with that obtained using 0.5 mg/cm^2^ PtC (60%). The hydrogen flowrate produced when using the MEA covered with 1.5 mg/cm^2^ IrRuOx and 1.5 mg/cm^2^ at the anode side is less than that of commercial MEA. Hydrogen production is lower for commercial MEA due to the high voltage and ohmic loss [29]. A reduction of hydrogen ions through the membrane occurred and resulted in a decrease in the rates of electrochemical reactions.

In terms of energy consumption, the MEA that has a low catalyst loading of 0.5 mg/cm^2^ PtC (60%) exhibited a high consumption. The high voltage obtained from this MEA caused an increase in energy consumption due to the directly proportional relationship between energy consumption and cell voltage. Based on Figure 11b, the energy consumption for commercial MEA and the MEA covered with 1.5 mg/cm^2^ IrRuOx and 1.5 mg/cm^2^ PtB are nearly the same. When the current density was less than 300 mA/cm^2^, the MEA with 1.5 mg/cm^2^ IrRuOx anode electrode catalyst and 1.5 mg/cm^2^ PtB recorded a higher energy consumption than commercial MEA due to higher cell voltage. However, its energy consumption is lower than that of commercial MEAs when the current density was high (>300 mA/cm^2^) due to the increased difference in hydrogen flowrates between the two MEAs. Thus, the 3.0 mg/cm^2^ IrRuOx is the optimal catalyst because it uses the least energy. For all cases in Figure 11b, the optimal current density is 200 mA/cm^2^, which represents the lowest energy consumption.

**Figure 12:**
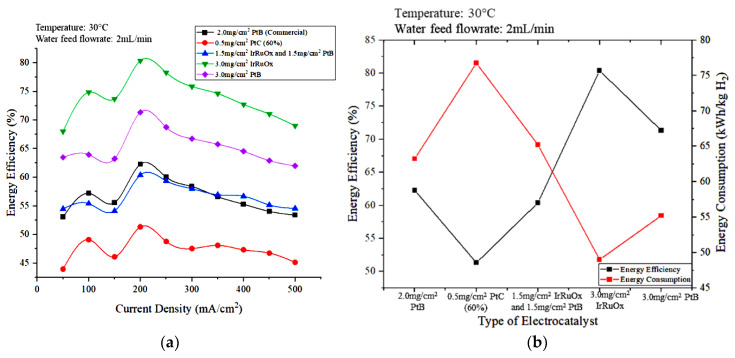
Effects of electrode types on (**a**) the energy efficiency versus current density and (**b**) energy efficiency and energy consumption at 200 mA/cm^2^.

Figure 12a,b show the effect of the type and loading of the anode electrocatalyst on energy efficiency and the energy efficiencies and energy consumption of different electrocatalyst types, respectively, at a current density of 200 mA/cm^2^. The electrode catalyst with 3.0 mg/cm^2^ IrRuOx exhibited the highest energy efficiency, followed by 3.0 mg/cm^2^ PtB. The electrical energy consumption of the 3.0 mg/cm^2^ IrRuOx is lower than that of 3.0 mg/cm^2^ PtB, which leads to higher energy efficiency for the PEM electrolyzer. The energy efficiency of 0.5 mg/cm^2^ PtC (60%) was the lowest because the low metal loading increased overpotential. Thus, the degradation rate increased with decreasing anode load [30].

Based on Figure 12, using 3.0 mg/cm^2^ IrRuOx as the electrode of the anode part resulted in low energy consumption with high energy efficiency and hydrogen production rate. Moreover, energy efficiency of 80.38% and energy consumption of 49 kWh/kg H_2_ was achieved. The operating temperature was set at 30 °C during the study for safety purposes. The same trend is expected to be obtained if the optimum temperature of 80 °C was used. Overall, the 3.0 mg/cm^2^ IrRuOx catalyst was the most suitable electrode catalyst type to use to achieve the optimal conditions in this study.

## 4. Conclusions

This study focused on the effects of operating conditions on PEM electrolyzer performance. The effects of the power supply (50 mA/cm^2^ to 500 mA/cm^2^ range), water flowrate (0.5 mL/min to 5 mL/min), operating temperature (25 to 80 °C), and four different types of anode electrode catalysts were determined. The results show that optimal conditions can be achieved when water is fed at a rate of 2 mL/min at a temperature of 70 °C, possibly due to improved kinetics of the charge-transfer reaction as well as increased membrane conductivity. These phenomena can result in a lower ohmic overvoltage and reduce the charge-transfer resistance on both sides of the electrode. Thus, the electrocatalytic reaction rates of hydrogen evolution and oxygen evolution are increased at both electrodes, increasing the voltaic efficiency of the electrolyzer. The low energy consumption of the PEM electrolyzer leads to an increased energy efficiency of up to 80%. Electrodes with the 3.0 mg/cm^2^ IrRuOx catalyst are most suitable for use due to their high conductivity and absence of MEA degradation issues. All these results indicate the importance of determining the effects of the operating parameters and types of anode electrocatalysts on the performance and durability of PEM electrolyzers to expand their application.

## Figures and Tables

**Table 1:** MEA Specifications.

Characteristic	Specification
Active area	6.0 cm × 6.0 cm
Electrolyte membrane	Nafion^®®^ 115
Electrocatalyst at the cathode side	3.0 mg/cm^2^ Platinum Black (PtB)
Electrocatalyst at the anode side	0.5 mg/cm^2^ Platinum Carbon (PtC) (60%) 1.5 mg/cm^2^ Iridium Ruthenium Oxide (IrRuOx) and 1.5 mg/cm^2^ Platinum Black (PtB) 3.0 mg/cm^2^ Iridium Ruthenium Oxide (IrRuOx) 3.0 mg/cm^2^ Platinum Black (PtB)

## Data Availability

All relevant data are contained in the present manuscript.
